# Identifying SLC27A5 as a potential prognostic marker of hepatocellular carcinoma by weighted gene co-expression network analysis and in vitro assays

**DOI:** 10.1186/s12935-021-01871-6

**Published:** 2021-03-17

**Authors:** Fan Zhang, Mengjuan Xue, Xin Jiang, Huiyuan Yu, Yixuan Qiu, Jiaming Yu, Fan Yang, Zhijun Bao

**Affiliations:** 1 Department of Geriatric Medicine, Huadong Hospital, Shanghai Medical College, Fudan University, No. 221 Yan’an West Road, Shanghai, 200040 People’s Republic of China; 2 Shanghai Key Laboratory of Clinical Geriatric Medicine, Huadong Hospital, Shanghai Medical College, Fudan University, No. 221 Yan’an West Road, Shanghai, 200040 People’s Republic of China; 3 Research Center on Aging and Medicine, Fudan University, Shanghai, 200040 People’s Republic of China

**Keywords:** Hepatocellular carcinoma, WGCNA, SLC27A5, Migration, Epithelial–mesenchymal transition

## Abstract

**Background:**

The incidence and mortality rates of hepatocellular carcinoma are among the highest of all cancers all over the world. However the survival rates are relatively low due to lack of effective treatments. Efforts to elucidate the mechanisms of HCC and to find novel prognostic markers and therapeutic targets are ongoing. Here we tried to identify prognostic genes of HCC through co-expression network analysis.

**Methods:**

We conducted weighted gene co-expression network analysis with a microarray dataset GSE14520 of HCC from Gene Expression Omnibus database and identified a hub module associated with HCC prognosis. Function enrichment analysis of the hub module was performed. Clinical information was analyzed to select candidate hub genes. The expression profiles and survival analysis of the selected genes were performed using additional datasets (GSE45267 and TCGA-LIHC) and the hub gene was identified. GSEA and in vitro experiments were conducted to further verify the function of the hub gene.

**Results:**

Genes in the hub module were mostly involved in the metabolism pathway. Four genes (SLC27A5, SLC10A1, PCK2 and FMO4) from the module were identified as candidate hub genes according to correlation analysis with prognostic indicators. All these genes were significantly down-regulated in tumor tissues compared with non-tumor tissues in additional datasets. After survival analysis and network construction, SLC27A5 was selected as a prognostic marker. GSEA analysis and in vitro assays suggested that SLC27A5 downregulation promoted tumor cell migration via enhancing epithelial-mesenchymal transition.

**Conclusion:**

SLC27A5 is a potential biomarker of HCC and SLC27A5 downregulation promoted HCC progression by enhancing EMT.

**Supplementary Information:**

The online version contains supplementary material available at 10.1186/s12935-021-01871-6.

## Background

Liver cancer is one of the most prevalent malignancies worldwide ranking as the sixth leading cause for cancer incidence and forth for modality [1]. Among all cases, 75–85% are hepatocellular carcinoma (HCC). Current curative treatments for HCC include liver resection, orthotopic liver transplantation and local destruction methods for early stage diseases [2]. However, more than 50% of HCC cases are in advanced stages and are not eligible for curative therapies at the time of diagnosis [3] and systemic treatment options are quite limited. The small-molecule multi-kinase inhibitors sorafenib and other targeted therapies has been applied in unresectable HCC but only provide a modest survival benefit of no more than 4 months compared with placebo [4–7]. The prognosis of the disease is quite poor with the overall 5-year survival rate < 20% [8] largely due to high incidence of recurrence and metastasis. Despite great efforts have been made to reveal the mechanism of the initiation and progression of HCC, we do not yet have a comprehensive understanding of the disease due to its complexity. And there is an urgent need for the identification of novel therapeutic targets and effective treatments nowadays.

Microarray and high throughout sequence data provide an opportunity to understand the landscape of changes in tumor genome. Bioinformatics mining may shed light on the exploration of new diagnostic and prognostic markers and offer cues for mechanism research. Weighted gene co-expression network analysis (WGCNA) is an effective tool for correlation network construction and hub gene identification [9] and is widely used in finding biomarkers for cancers. Highly correlated genes may be functionally related and can be clustered by WGCAN into a module. Correlations between modules with clinical features can be quantified and help identify modules of interest. The central nodes of a modules are regarded as the hub genes which represent the principle role of a network, and maybe, of a disease.

The incidence rates of HCC peak in the elderly almost in all areas [10]. It is more common in the aged, however, more devastating in the young [11]. Studies found that young HCC patients have more advanced tumor and lower survival rates compared with the elderly [12, 13]. If young HCC patients survive longer than one year, their prognosis will be better than the elderly due to better liver function reserves [13]. The transcription profile of young HCC patients is completely distinct from that of the elderly and show increased embryonic stem cell traits [11]. The study by Langhans [14] found inverse correlations between age of HCC patients (older than 50 years in this study) and frequency of peripheral Tregs expressing PD-L1 (*r*^2^ = − 0.1843, *P* = 0.0080) and CTLA-4 (*r*^2^ = − 0.2265, *P* = 0.0029). In Asia, HBV infection is one of the predominant causes of hepatic cirrhosis and HCC. In this study a microarray dataset of mainly HBV positive patients were used to investigate the mechanisms of HCC and to identify prognostic markers by WGCNA. Co-expression network was constructed and the hub module and hub gene were identified according to their correlations with prognostic indicators. We also paid attention to the expression level of candidate genes in different age groups. The hub gene was validated by additional datasets and its role in HCC pathogenesis was interrogated by in vitro assays.

## Materials and methods

### Data acquiring and processing

The hepatocellular carcinoma microarray dataset GSE14520 provided by Wang et al. [15] (https://www.ncbi.nlm.nih.gov/geo/query/acc.cgi?acc=GSE14520) was obtained from the Gene Expression Omnibus (GEO) database. This dataset contained 247 predominantly HBV-positive cases carried out on the platform of HG-U133A_2 (GPL571) for training group and HT_HG-U133A (GPL3921) for testing group, respectively. The raw data and clinical information were downloaded. Files in.CEL format were normalized with Robust Multi-array Average (RMA) method. Expression matrixes of the two platforms were batched by SVA package. We acquired the annotation information by R package hgu133a2.db for GPL571 and hthgu133a.db for GPL3921, respectively. We selected the maximum value for genes corresponding to more than one probe.

### Differentially expressed genes screening

The limma package was used to screen the DEGs between HCC tumor tissues and non-tumor tissues. Genes with adj.*P* < 0.05 and |log2 fold change|> 1 were considered as DEGs.

### Establishment of co-expression network

The tumor samples of the 247 patients were used for the co-expression network analysis. The variance of every gene among 247 samples was calculated. Top 25% of the most variant genes (3101 genes) were selected as candidate genes. We used the R package “WGCNA” to perform step-by-step network construction and the network type was “unsigned” [9]. The soft-thresholding power β was determined based on the criteria of approximate scale free topology. The power was used to define the adjacency matrix which was then transformed into topological overlap matrix (TOM). Next, we performed gene clustering analysis with TOM. Assignment of the candidate genes into different modules was accomplished using dynamic tree cutting method.

### Relating modules to clinical features and identifying the module of interest

Module eigengene (ME) is summarized by the first principal component of a module and is used to represent the expression profile of the module. The correlations of MEs with clinical parameters were calculated to quantify module-trait associations. The module that most significantly correlated with the prognostic indicators would be the module of interest. The network of the hub module was exported to Cytoscape 3.7.0 and the threshold of topological overlap was set to 0.04. The network analyzer tool of Cytoscape was used to analyze and visualize the main network of the hub module.

### Gene ontology (GO) enrichment and Kyoto encyclopedia of genes and genomes (KEGG) pathway analysis of the hub module

GO enrichment and KEGG pathway analysis of the hub module was performed using KOBAS 3.0 [16] online tools. Overrepresentation analysis (ORA) based on the hypergeometric distribution was used as the gene set enrichment method. The significantly enriched terms containing the largest numbers of genes were visualized by R package ggplot2.

### Identification and validation of hub genes

The module membership (MM) is defined as the correlation of ME with the gene expression profile, which quantifies how close the gene is to a given module. Gene significance is the correlation between the gene expression values and a specific clinic trait. Genes with both high MM and GS were considered as the candidate hub genes. Raw data of the dataset GSE45267 [11] (https://www.ncbi.nlm.nih.gov/geo/query/acc.cgi?acc=GSE45267) were downloaded to validate the expression levels of hub genes in tumor and non-tumor tissues. The process of raw data was the same as that of GSE14520. The transcriptome profiling and clinical information of the TCGA-LIHC project were downloaded from the GDC Data Portal (https://portal.gdc.cancer.gov). The online database GEPIA (http://gepia.cancer-pku.cn/index.html) [17] was used to perform survival analysis of hub genes based on the TCGA-LIHC project. We further performed survival analysis of hub genes on HBV positive patients of the TCGA-LIHC project. HBV infection was diagnosed if anyone of the following indicators were positive: HBV surface antigen; HBV DNA; HBV Core antibody. According to this criteria, 144 patients of the TCGA-LIHC project were HBV positive. Among them, 140 patients with intact follow-up information were included in the survival analysis.

### Gene set enrichment analysis

Gene set enrichment analysis (GSEA) was performed by the software GSEA 4.1.0 [18, 19]. A total of 247 tumor samples in GSE14520 were divided into high expression and low expression subgroups according to the median expression level of the hub gene. “c2.cpg.v7.0.symbols.gmt” and “h.all.v7.1.symbols.gmt” (MSigDB, http://software.broadinstitute.org/gsea/msigdb/index.jsp) were used as the reference gene sets.

### Cell culture

Human liver cancer cell line HepG2 was obtained from the FuHeng Biology (Shanghai, China). Eagle’s Minimum Essential Medium (EMEM) (Gibco, Australia) supplemented with 10% fetal bovine serum (Gibco, Australia) was used for the culture of HepG2 cells. Cells were cultured in a humidified environment consisting of 95% air and 5% CO_2_ at 37 °C.

### Cell transfection

The sequences of SLC27A5 small interference RNA were as follows: 5′-GCGUGACAGUGAUCCUGUAdTd-3′ and 5′-UACAGGAUCACUGUCACGCdTd-3′. The siRNAs were transfected into cells using Lipofectamine RNAiMAX (Thermo Fisher, USA) according to the manufacturer’s instruction. RNA and protein samples were collected 48–72 h after transfection.

### RNA extraction and qPCR

Total RNA was extracted from cultured cells by TRIzol reagent (Invitrogen, USA) and were reverse-transcribed to cDNA by PrimerScript RT Master Mix (Takara, Japan). qPCR was performed using SYBR Green Premix Ex Taq (Takara, Japan) on a Stepone Plus Real-Time PCR system (Aplied Biosystem life technology). Primer sequences were as follows: SLC27A5 5′-CGTGATGGGACTTGTCGTTG-3′ and 5′-CGACAGTCATCCCAGAAGCA-3′; β-actin 5′-GGACTTCGAGCAAGAGATGG-3′ and 5′-AGGAAGGAAGGCTGGAAGAG-3′; 18s-RNA 5′-GTAACCCGTTGAACCCCATT-3′ and 5′-CCATCCAATCGGTAGTAGCG-3′; SOX2 5′-TGGACAGTTACGCGCACAT-3′ and 5′-CGAGTAGGACATGCTGTAGGT-3′; MMP7 5′-GAACAGGCTCAGGACTATCTCA-3′ and 5′-GGCTTCTAAACTGTTGGCATT-3′; IL8 5′-AAGGAAAACTGGGTGCAGAG-3′ and 5′-ATTGCATCTGGCAACCCTAC-3′. The PCR parameters were: 95 °C 30 s, followed by 40 cycles of 95 °C 5 s and 60 °C 30 s. For the relative gene expressions, the values were normalized to β-actin levels as ΔCt and then transferred to the fold change (2^−ΔΔCt^) by comparing them with the negative control.

### Western blot anaylsis

Cells were lysed with the protein extraction reagent RIPA (Beyotime, China) supplemented with 1% PMSF (Beyotime, China). Protein concentration was measured by a bicinchoninic acid protein assay kit (Thermo, USA). The protein samples were electrophoresed on SDS-PAGE gel and transferred to polyvinylidene fluoride membranes (Millipore, USA). After being blocked in 5% bovine serum albumin for 2 h at room temperature, the membranes were incubated with the following primary antibodies: FATP5 (1:1000, Abcam, #ab74869), E-cadherin (1:1000, CST, # 3195), Snail (1:1000, CST, # 3879), c-myc (1:1000, CST, #5605), and β-actin (1:2000, proteintech, China, #HRP-66009) at 4 °C overnight. The next day the membranes were incubated with horseradish peroxidase-labeled goat anti-rabbit secondary antibody (1:2000, CST, #7074P2) for 1 h at room temperature. An ECL chemiluminescence kit (Thermo, USA) was used to detect specific bands.

### Transwell assay

Cell migration assays were performed using transwell chambers (24-well insert, 8 μm, Corning Costar Corp). Cells were harvested at 72 h after transfection. 1 × 10^5^ cells were suspended in 100 µl serum-free medium and placed in the upper chamber. The lower chamber was filled with 600 µl of medium containing 20% fetal bovine serum. After incubation for 24 h at 5% CO_2_ at 37 °C, the cells remaining on the upper chamber were removed with cotton wool, cells migrating to the bottom surface of the membrane from the upper chamber were fixed with 4% paraformaldehyde, stained with 0.1% crystal violet solution, then were imaged and four fields were counted.

## Results

### Data processing

The raw data of 247 tumor and 239 non-tumor samples of GSE14520 were downloaded from GEO and were processed by RMA algorithm. 12,402 non-duplicated probes were screened for DEGs between tumor and non-tumor tissues. The results were shown in Additional file 1: Figure S1. We used the data of 247 tumor samples to construct co-expression network. The most variant 3101 genes were selected for co-expression analysis.

**Fig. 1:**
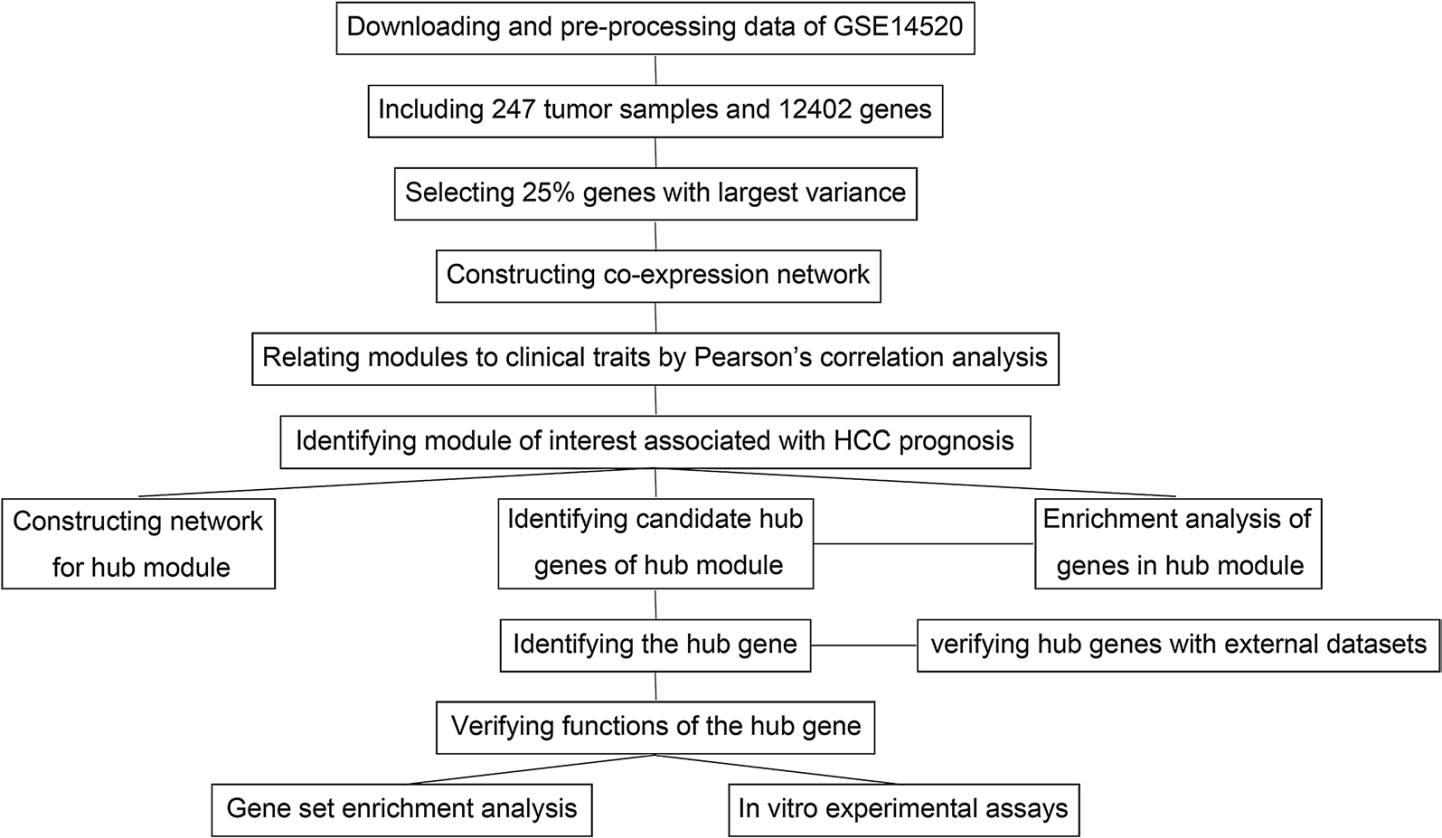
The flowchart of the study

The workflow of the study is presented in Fig. 1.

### Constructing weighted co-expression network

**Fig. 2:**
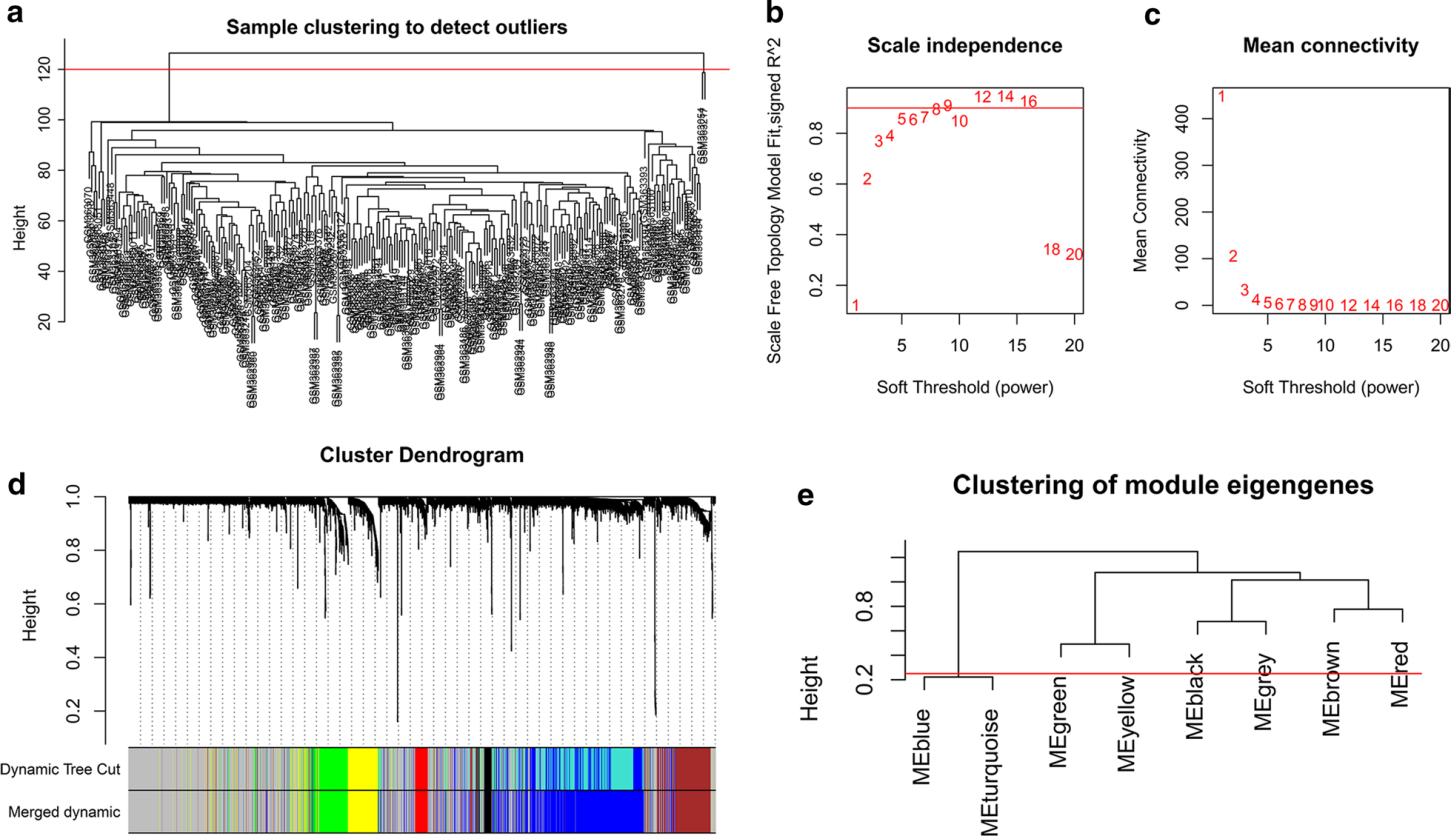
Co-expression network construction. **a** Clustering dendrogram of samples. Two outliers were excluded before subsequent analysis. The red line represented the threshold for removing outliers. **b** The scale-free fit index for soft-thresholding powers of 1–20. **c** The mean connectivity for 1–20 soft-thresholding powers. **d** Gene clustering dendrogram generated based on topological overlap matrix, together with the assigned merged module colors and the original colors. **e** Modules clustering dendrogram based on correlations of module eigengenes. The red line represented the merging threshold (0.25). Modules below this line would be merged

The cluster analysis of selected samples was shown in Fig. 2a and two outliers were excluded from subsequent analysis. The soft-thresholding power was set to 8 when the scale free topology fit index reached 0.9 (as shown in Fig. 2b, c). The adjacency matrix was calculated with the power 8 and was turned into TOM. A hierarchical clustering tree (dendrogram) of genes was produced using TOM. In the clustering tree, each leaf corresponded to a gene. Highly co-expressed genes grouped together and formed a branch of the tree. The dynamic tree cut method was used for branch cutting and each branch represented a module (as shown in Fig. 2d). We calculated MEs and selected a height cut of 0.25 for merging of modules whose MEs correlated with each other above 0.75. Cluster analysis of entire modules based on correlations of MEs showed 8 original modules were grouped into three clusters (Fig. 2e). The blue and the turquoise modules were merged into one under the height cut of 0.25. Genes that did not belong to any module were assigned to the grey module. 6 modules were generated finally except for the grey module (Table 1).

**Table 1:** Numbers of genes for six modules

Module	Genes
Blue	853
Brown	312
Red	68
Black	54
Green	235
Yellow	260

### Identification of the hub module

**Fig. 3:**
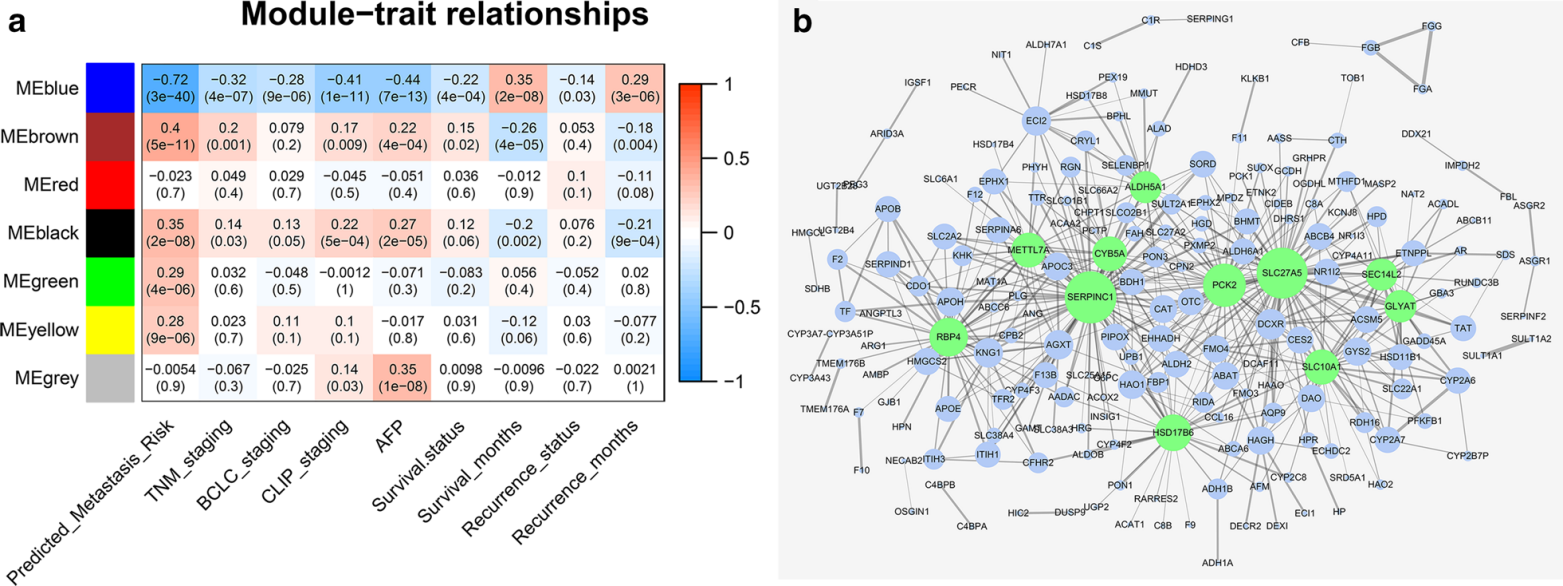
Identification of the hub module. **a** Heatmap of correlations of module eigengenes with clinical traits. Each row corresponded to a module eigengene, column to a clinical trait. The numbers in each cell represented corresponding correlation and *P* value. The table was color-coded by correlation according to the color legend. **b** Network of genes in the blue module. The size of the node correlated with the degree of connectivity and the width of the edge with weight. The green nodes represented the central nodes with more than 20 connections

The associations between modules and prognosis of HCC were qualified by Pearson correlation between MEs and clinical traits and were shown in heatmap in Fig. 3a. A metastasis-related gene signature based on expression profiles of HCC samples was developed by Ye et al. [20] and was validated by the current dataset [15]. The result was referred to as “predicted metastasis risk”. The BCLC [21] and CLIP [22] are commonly used staging systems for prognostic prediction and treatment decision and the higher the staging, the poorer the prognosis. The blue module were negatively correlated with predicted metastasis risk, TNM staging, BCLC staging, CLIP staging, AFP level, death and recurrence and positively correlated with survival months and recurrence free survival months. The brown and black module were almost the opposite. The green and yellow were positively correlated with predicted metastasis risk. Other correlations were not significant under the *P* value of 0.05. Subsequently, we focused on the blue module which had higher absolute correlations with all the prognostic indicators. The blue module consisted of 853 genes including 55 significantly up-regulated genes and 312 down-regulated genes. Network of the blue module based on similarity of TOM was exported and the threshold of topological overlap was 0.04. The network was visualized by Cytoscape 3.7.0 based on degree of connectivity. The main network was composed of several subnets. Nodes with 20 or more connections were marked in green and they were also centers of different subnets (Fig. 3b).

### GO function and KEGG pathway analysis of the hub module

**Fig. 4:**
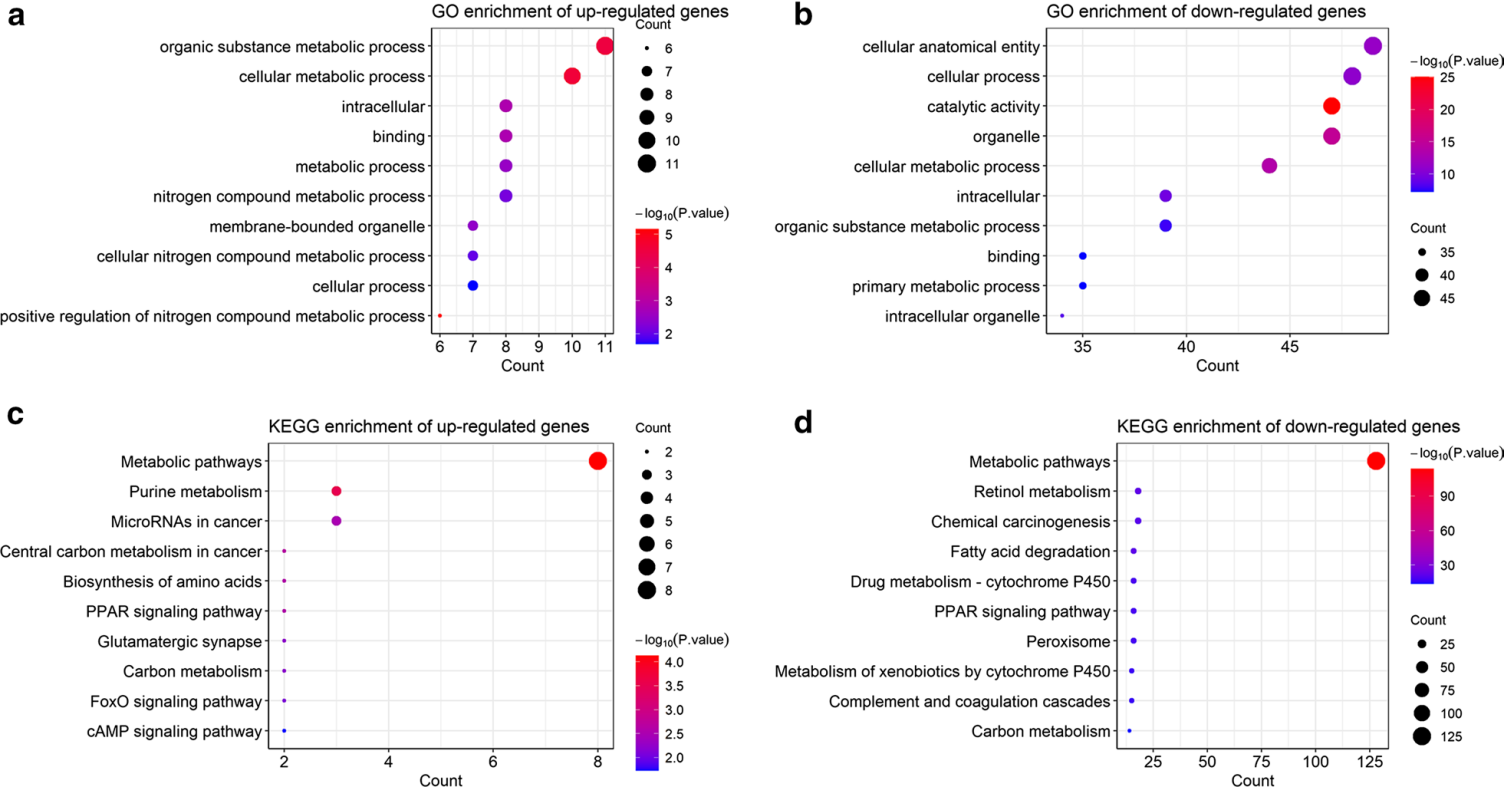
Enrichment analysis of DEGs in the blue module according to the fold change. The left panel was the results of GO (**a**) and KEGG (**c**) enrichment analysis of the up-regulated genes. The right panel was that of the down-regulated genes (**b**, **d**). The terms were ranked by gene count in descending order. The size of the dots indicated the number of genes enriched in corresponding term

GO and KEGG analysis of DEGs in the blue module were performed according to their fold change and the results were shown if Fig. 4. Notably, both the up-regulated and down-regulated groups of genes were enriched in cellular metabolic process by GO analysis (Fig. 4a, b) and metabolism pathways, PPAR signaling pathways and carbon metabolism by KEGG analysis (Fig. 4c, d). Other enriched terms of up-regulated genes were nitrogen compound metabolic process (Fig. 4a), biosynthesis of amino acids by KEGG (Fig. 4c). The down-regulated genes were enriched in retinol metabolism, fatty acid degradation and cytochrome 450 by KEGG (Fig. 4d).

### Identification of the hub genes

**Fig. 5:**
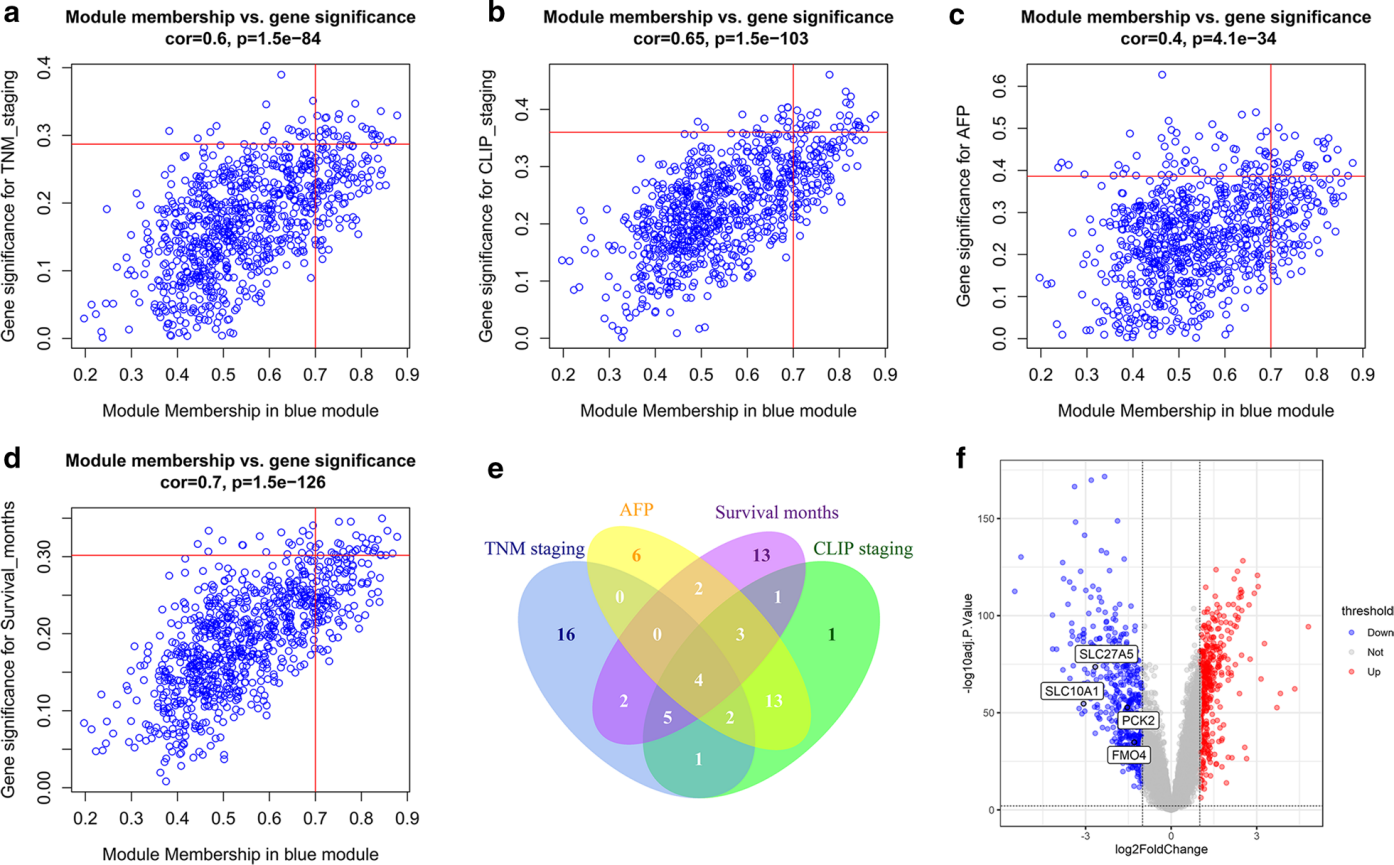
Identification of hub genes. Scatterplots of absolute gene significance (GS) for TNM staging (**a**), CLIP staging (**b**), AFP level (**c**) and survival months (**d**) vs. absolute module membership (MM) in the blue module. Genes in the upper right quadrant of each plot were candidate hub genes. The cutoffs were MM > 0.7 and GS ranked top 30. Hub genes were identified by intersection of four groups of candidate hub genes (**e**) and were labeled on the volcano plot of DEGs (**f**)

Subsequently, we calculated the gene significance (GS) of the blue module for clinical features with an absolute correlation greater than 0.3, including TNM staging, CLIP staging, AFP, and survival months (Fig. 5a). We did not include the predicted metastasis risk as it was based on gene expression profile, not actually observed. The correlations between absolute GS with absolute MM were calculated and presented by scatterplots in Fig. 5a–d. Hereafter, when we mentioned GS and MM, we were referring to their absolute values. The correlations between GS and MM were high in the blue module and demonstrated that genes with high MM also had high GS for corresponding clinical traits. For each clinical trait, genes with MM greater than 0.7 and ranked in the top 30 in GS were regarded as candidate hub genes (as shown in the upper right quadrant of Fig. 5a–d). The intersection of the four groups of candidate hub genes contained SLC27A5, SLC10A1, PCK2 and FMO4 (Fig. 5e). The four genes were labeled in the volcano plot of DEGs (Fig. 5f). The expression of hub genes were significantly lower in HCC tumor tissues than in normal tissues. The |log2 fold change| of SLC27A5 and SLC10A1 was more than 2.5.

### Validating hub genes by additional datasets

**Fig. 6:**
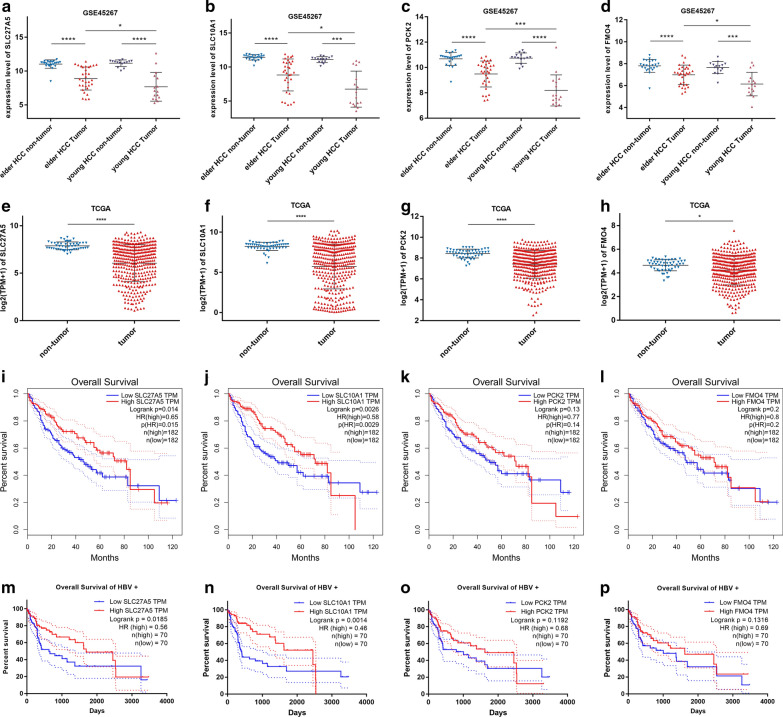
Differential expression and survival analysis of hub genes based on dataset GSE45267 and TCGA-LIHC. Relative expression levels of SLC27A5, SLC10A1, PCK2 and FMO4 in non-tumor and tumor tissues from GSE45267 (**a**–**d**) and TCGA-LIHC (**e**–**f**) compared by Mann–Whitney test. The overall survival time of all patients (**i**–**l**) and HBV positive patients (**m**–**p**) in TCGA-LIHC grouped by the expression level of SLC27A5, SLC10A1, PCK2 and FMO4. *P < 0.05, ***P < 0.001, ****P < 0.0001

In the dataset GSE45267 (Fig. 6a–d) and the TCGA project LIHC (Fig. 6e–h), the relative expression profile of the hub genes between tumor and non-tumor tissues were consistent with the current dataset. Besides, in GSE45267, the four genes were slightly but significantly lower in young HCC tumor than in elder HCC tumor tissue (Fig. 6a–d). Although HCC is more common in the elderly, it is more malignant in young patients than in older patients [11]. Survival analysis was performed on all patients (Fig. 6i–l) and 140 HBV positive patients (Fig. 6m–p) of the TCGA-LIHC cohort by Logrank test. The former analysis was conducted on the website of GEPIA. Only the expression levels of SLC27A5 and SLC10A1 were positively associated with longer overall survival time both in all patients and in HBV positive patients (Fig. 6i–p). We selected SLC27A5, the second largest center of the network (Fig. 3b), as a prognostic biomarker for further research.

### Knockdown of SLC27A5 significantly promoted cell migration

**Fig. 7:**
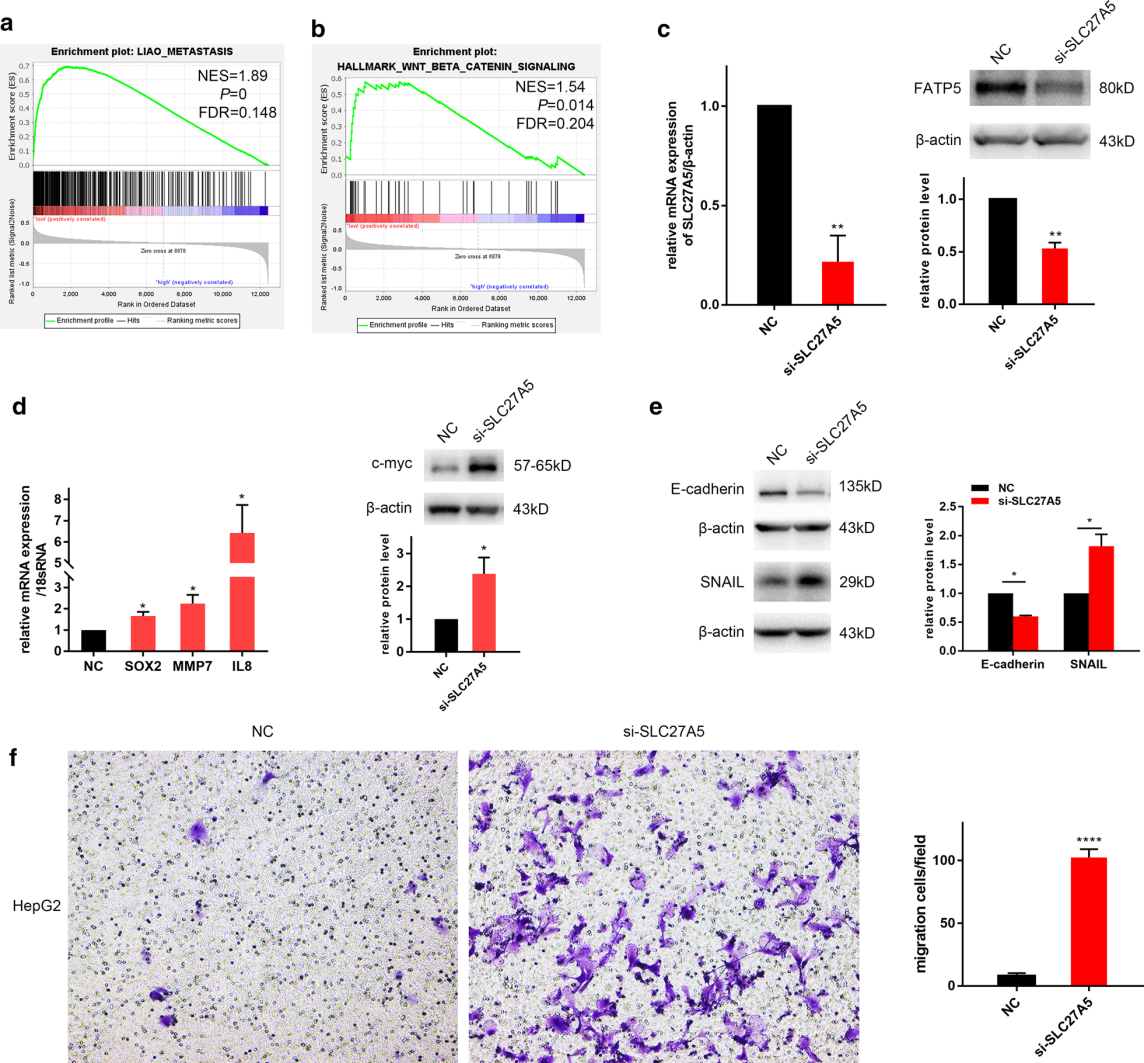
GSEA and in vitro experiments in HepG2 cell line. The gene set of LIAO_METASTASIS (**a**) and Wnt/β-catenin pathway (**b**) was significantly enriched in the low SLC27A5 group. qPCR and western blot showed FATP5 (SLC27A5) in HepG2 was knocked down by small interference RNA (**c**). Both experiments were replicated three times. ** *P* < 0.01 by paired Student’s t test. The level of SOX2, MMP7, IL8 by qPCR and c-myc by western blot all increased after SLC27A5 knocked down (**d**). The tests were replicated three times, respectively. *P < 0.05 by paired Student's t test (**e**) Western blot assasys indicated that epithelial mesenchymal transition markers were altered after SLC27A5 knocked down. The assays were replicated at least twice. * *P* < 0.05 by paired Student’s t test. (**f**) The transwell migration assay showed increased migration ability of HepG2 cells treated with si-SLC27A5. Four fields were captured at 100 × magnification. **** *P* < 0.0001 by Student’s t test

We used GSEA to explore the function of the hub gene. Tumor samples of GSE14520 were divided into low expression and high expression subgroups according to the median expression level of SLC27A5. The high expression group was used as the control. GSEA showed that two gene sets, LIAO_METASTASIS and Wnt/β-catenin pathway were significantly enriched in the low expression group (Fig. 7a, b). The LIAO_METASTASIS is a set of genes up-regulated in the samples with intrahepatic metastatic HCC versus primary HCC [23]. Wnt/β-catenin is closely related with epithelial-mesenchymal transition (EMT) and is involved in tumor metastasis. So we supposed that SLC27A5 downregulation in tumor tissues might be associated with cancer metastasis. To verify this hypothesis, small interference RNAs were used to lower SLC27A5 expression in HepG2 cells. The knockdown efficiency was determined by qPCR and the level of FATP5 (protein encoded by SLC27A5) was detected by western blot (Fig. 7c). Several targets of Wnt/β-catenin pathway [24], including SOX2, MMP7, IL8 and c-myc, all increased after SLC27A5 knockdown (Fig. 7d). The expression of E-cadherin was decreased while Snail was increased by si-SLC27A5 (Fig. 7e), indicating that cells were in the process of EMT. In the transwell assay, the migration ability of HepG2 increased significantly after SLC27A5 knockdown (Fig. 7f).

## Discussion

Despite the incidence rate is decreasing in several regions [25], HCC is still one of the leading causes of cancer related death worldwide with poor prognosis. Intra and extra hepatic metastasis is the main reason for advanced staging and treatment failure of HCC. Identifying molecular markers associated with clinical outcome would be beneficial for mechanism research and the discovery of novel therapeutic targets. In this study, we analyzed the HCC dataset GSE14520 by WGCNA and identifying the hub module according to the correlation with prognostic indicators. We selected SLC27A5 as a prognostic gene of HCC and further in vitro assays suggested that it promoted tumor migration via enhancing EMT.

We identified the blue module as the hub module that highly correlated with HCC prognostic indicators. Enrichment analysis demonstrated that the up- and down- regulated DEGs in the blue module were all enriched in cellular metabolic process and metabolic pathways (Fig. 4). Cancer metabolic reprogramming has been gaining increasing interest in the past decades. One of its hallmarks is increased demand for carbon and nitrogen to build diverse biomass and compounds [26]. Here we showed that the up-regulated genes in the blue module were significantly enriched in carbon metabolism (Fig. 4a) and nitrogen compound metabolic process (Fig. 4c), so as the down-regulated genes in carbon metabolism (Fig. 4d). Another common enriched term of the two groups of DEGs was PPAR signaling pathway (Fig. 4c, d). PPARs are a family of nuclear receptors that plays an important role in energy metabolism, cellular development and inflammation. Studies have addressed the pleiotropic effects of PPARs in tumorigenesis as they can either promote or inhibit tumor growth [27]. However, the scenario is still ambiguous, and the application of PPAR-targeted drugs in cancer treatment should be with caution [28].

We also found that the down-regulated genes in the blue module were enriched in the retinol metabolism (Fig. 4d). Retinoids participate in various biological processes including cell growth, development, differentiation and apoptosis. Dysregulation of retinoids and relevant pathways have been observed in animal tumor models and human carcinoma cell lines [29]. Retinoids deficiency is associated with increased risk of carcinogenesis [30–32] and with poor prognosis of liver cancer [33]. Briefly, our data showed that metabolic pathway was highly associated with the prognosis of HCC. Metabolic reprogram is one of the hallmarks of cancer [34] including changes in biological macromolecules and small molecule metabolites. Increasing metabolic mechanism have been reported to be involved in the progression of cancer. The hub gene of our study SLC27A5 (FATP5) is a multifunctional enzyme involved in the metabolism of fatty acids and bile acids.

FATP5 is a liver-specific member of fatty acid transport family [35]. In addition to its role in facilitating fatty acid transport, FATP5 is involved in the activation of very long-chain fatty acids and reconjugation of bile acids during the enterohepatic circulation [36]. FATP5 deletion resulted in lower hepatic triglyceride and free fatty acid content and protected mice from obesity induced by high fatty diet [36, 37]. While previous studies focused on the function of SLC27A5 in lipid metabolism and metabolism syndrome [38, 39], Gao et al. [40] investigated the role of SLC27A5 in HCC for the first time. According to their study, SLC27A5 deficiency promotes tumor proliferation and chemoresistance by increasing 4-HNE level and consequently activating KEAP1/NRF2/TXNRD1 pathway. The underlying mechanism is that knockout of SLC27A5 increases polyunsaturated lipids, leading to reactive oxygen species (ROS) production, lipid peroxidation and accumulation of 4-HNE. Their study revealed the function of SLC27A5 in the maintenance of redox homeostasis. In the current study, knockdown of SLC27A5 activated targets of Wnt/β-catenin pathway, promoted EMT and increased the migration ability of liver tumor cells. We suppose the pro-migrating effect of SLC27A5 knockdown is also due to the increase in ROS production. ROS is involved in ionizing radiation induced EMT [41]. In pancreatic ductal adenocarcinoma, ROS drived EMT by activating Akt/glycogen synthase kinase 3β (GSK3β)/Snail signaling [42]. Another study demonstrated that ROS facilitated EMT by inducing nuclear translocation of NRF2 to activate Notch pathway in HCC [43]. The mechanism of SLC27A5 downregulation to promote EMT and whether it is associated with ROS production warrant further research.

EMT contributes to nearly all of the hallmarks of cancer including proliferation, invasion, cancer stem cell properties and immune escape; it is associated with tumor metastasis, recurrence, and therapy resistance [44]. It is a formidable obstacle and an attractive target for cancer therapy [44]. We identified SLC27A5 as a prognostic marker of HCC by constructing a co-expression network and correlating modules and genes with detailed clinical indicators. Previous study reported its role in inhibiting proliferation and chemoresistance in HCC. We provided evidence for downregulation of SLC27A5 facilitating liver tumor cells migration by driving EMT. Metabolic changes of fatty acids and bile acids may be involved in the process.

In conclusion, we identified SLC27A5 as a prognostic marker of HCC by co-expression network construction and clinical information analysis. SLC27A5 was positively correlated with prognosis of HCC and was downregulated in tumor tissues compared with non-tumor tissues. Our in vitro experiments demonstrated SLC27A5 downregulation promoted HCC progression by driving EMT. Although the mechanism needs further research, we proposed new function of SLC27A5 and provided preliminary evidence, which may offer clues for subsequent studies.

## Supplementary Information

**Additional file 1.** Volcano plot displayed 430 up‐regulated and 508 down‐regulated DEGs of tumor tissues in the dataset GSE14520 compared with non-tumor tissues.

## Data Availability

The data supporting the findings of this study are available in GEO at https://www.ncbi.nlm.nih.gov/geo/, reference number GSE14520 and GSE45267, and in TCGA at https://portal.gdc.cancer.gov/, reference number TCGA-LIHC.

## Abbreviations

HCC: Hepatocellular carcinoma
WGCNA: Weighted gene co-expression network analysis
GEO: Gene expression omnibus
RMA: Robust multi-array average
DEGs: Differentially expressed genes
TOM: Topological overlap matrix
ME: Module eigengene
MM: Module membership
GO: Gene ontology
KEGG: Kyoto encyclopedia of genes and genomes
TCGA: The cancer genome atlas
LIHC: Liver hepatocellular carcinoma
GEPIA: The gene expression profiling interactive analysis database
BP: Biological processes
CC: Cell components
MF: Molecular functions
GSEA: Gene set enrichment analysis
