# Supplementary figures and images for: Identifying SLC27A5 as a potential prognostic marker of hepatocellular carcinoma by weighted gene co-expression network analysis and in vitro assays
Source: Cancer Cell Int. 2021 Mar 17;21:174. doi: 10.1186/s12935-021-01871-6 (PMC7968262; doi:10.1186/s12935-021-01871-6)

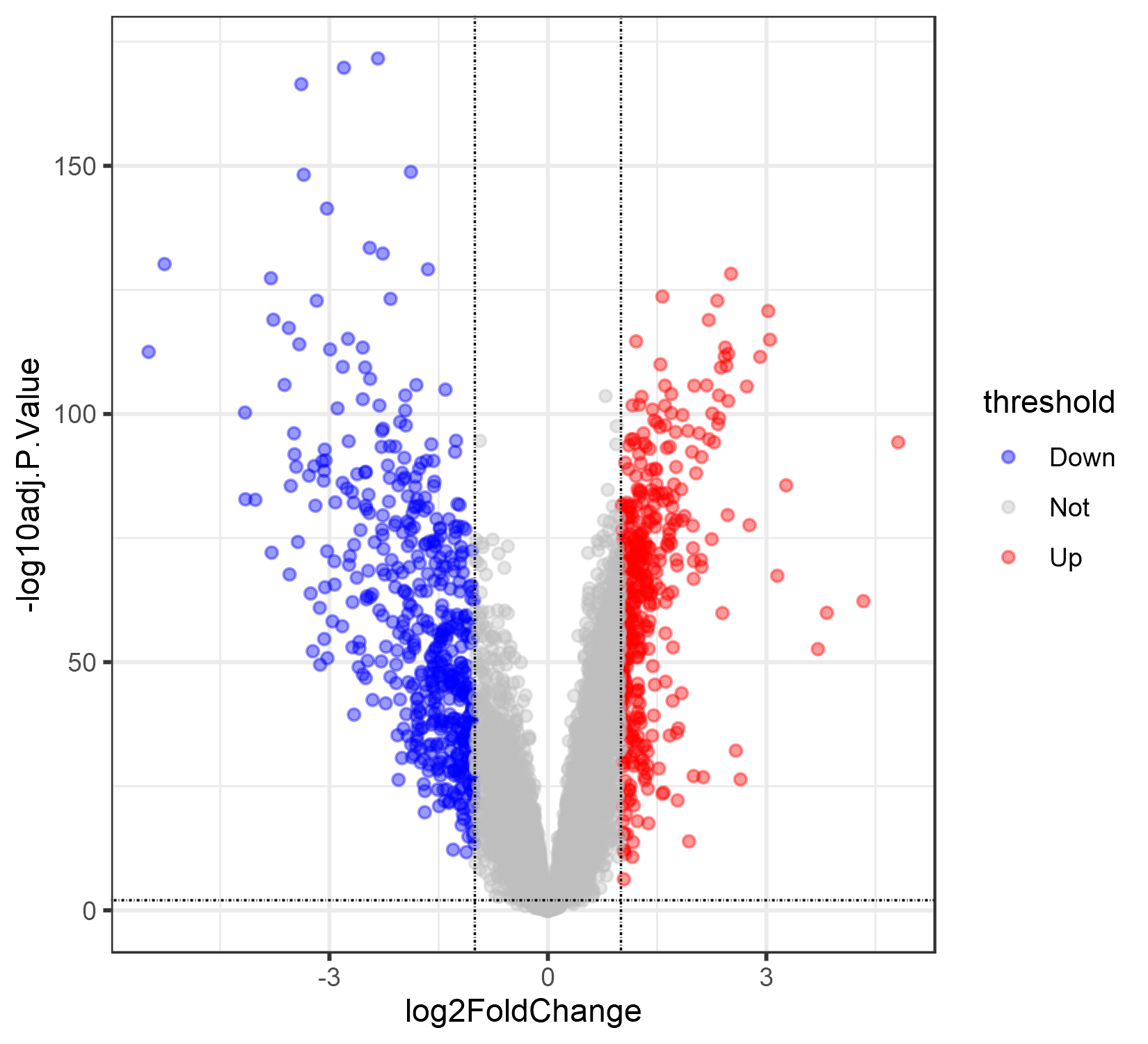

Supplement: Supplementary file 1 — Additional file 1. Volcano plot displayed 430 up‐regulated and 508 down‐regulated DEGs of tumor tissues in the dataset GSE14520 compared with non-tumor tissues. [file 12935_2021_1871_MOESM1_ESM.tif]
